# Radix Scrophulariae extracts (harpagoside) suppresses hypoxia-induced microglial activation and neurotoxicity

**DOI:** 10.1186/s12906-015-0842-x

**Published:** 2015-09-14

**Authors:** Shiow-Yunn Sheu, Yi-Wen Hong, Jui-Sheng Sun, Man-Hai Liu, Ching-Yun Chen, Cherng-Jyh Ke

**Affiliations:** School of Pharmacy, College of Pharmacy, Taipei Medical University, Taipei, Taiwan; Department of Orthopedic Surgery, National Taiwan University Hospital Hsin-Chu Branch, No.25, Lane 442, Sec. 1, Jingguo Rd., Hsin-Chu City, 30059 Taiwan; Department of Orthopedic Surgery, College of Medicine, National Taiwan University, No. 1, Sec. 1, Ren-Ai Rd., Taipei, 10051 Taiwan; Biomimetic Systems Research Center, National Chiao Tung University, Hsin-Chu, Taiwan; Department of Food Science, College Health Science and Technology, China University of Science and Technology, Taipei, Taiwan; Institute of Biomedical Engineering, College of Engineering and College of Medicine, National Taiwan University, Taipei, Taiwan

**Keywords:** Harpagoside, Hypoxia, Microglial cells, Activation, Anti-inflammation

## Abstract

**Background:**

Hypoxia could lead to microglia activation and inflammatory mediators’ overproduction. These inflammatory molecules could amplify the neuroinflammatory process and exacerbate neuronal injury. The aim of this study is to find out whether harpagoside could reduce hypoxia-induced microglia activation.

**Methods:**

In this study, primary microglia cells harvested from neonatal ICR mice were activated by exposure to hypoxia (1 % O_2_ for 3 h). Harpagoside had been shown to be no cytotoxicity on microglia cells by MTT assay. The scavenger effect of harpagoside on hypoxia-enhanced microglial cells proliferation, associated inflammatory genes expression (COX-II, IL-1β and IL-6 genes) and NO synthesis were also examined.

**Results:**

Hypoxia enhances active proliferation of microglial cells, while harpagoside can scavenge this effect. We find that harpagoside could scavenge hypoxia-enhanced inflammatory genes expression (COX-2, IL-1β and IL-6 genes) and NO synthesis of microglial cells. Under 3 h’ hypoxic stimulation, the nuclear contents of p65 and hypoxia inducible factor-1α (HIF-1α) significantly increase, while the cytosol IκB-α content decreases; these effects can be reversed by 1 h’s pre-incubation of 10^−8^ M harpagoside. Harpagoside could decrease IκB-α protein phosphorylation and inhibit p65 protein translocation from the cytosol to the nucleus, thus suppress NF-κB activation and reduce the HIF-1α generation.

**Conclusion:**

These results suggested that the anti-inflammatory mechanism of harpagoside might be associated with the NF-κB signaling pathway. Harpagoside protect against hypoxia-induced toxicity on microglial cells through HIF-α pathway.

## Background

The central nervous system (CNS) consists of both neurons and non-neuronal glial cells including microglia and astrocytes; microglia, the resident macrophages of brain, play critical roles in the maintenance of neural environment [1]. Microglia cells also appear to play an important role during normal function of the nervous system. Under diverse neurological conditions, such as stroke, Alzheimer’s disease (AD), Parkinson’s disease (PD), and nerve injury-induced neuropathic pain, or exposure to toxicological stimuli, microglia will be rapidly activated [2]. Activated microglial cells display macrophage-like characteristics including production of pro-inflammatory cytokines, antigen presentation and phagocytosis [3]. However, uncontrolled and sustained secretion of inflammatory cytokines by microglia also trigger neuronal cell death [4]. Inflammatory components related to neuro-inflammation include microglia and astrocytes, the complement system, as well as cytokines and chemokines [5].

In Alzheimer’s disease (AD), it has been observed in epidemiological studies that treatment with non-steroidal anti-inflammatory drugs (NSAIDs) decreases the risk for developing AD. Unfortunately, clinical trials of NSAIDs in AD patients have not been very fruitful. Proinflammatory responses may be countered through polyphenols. Supplementation of these natural compounds may provide a new therapeutic line of approach to this brain disorder [5]. Si-Miao-Yong-An (Trade name: Mai-Luo-Ning), a Chinese herbal formulation comprising Flos Lonicerae Japonicae, Radix Scrophulariae Ningpoensis, Radix Angelicae Sinensis and Radix Glycyrrhizae Uralensis, has been used in treating ischemic cardiovascular and cerebrovascular diseases for many years. Clinical and experimental studies have shown that Si-Miao-Yong-An can inhibit the inflammatory response and antagonize the blood clotting process. In the previous study, there was evidence that Si-Miao-Yong-An could promote the stability of atherosclerotic plaque in the rabbit model [6].

Preparations of Harpagophytum procumbens, known as devil’s claw, are used as an adjunctive therapy for the treatment of pain and osteoarthritis. Pharmacological evaluations have proven the effectiveness of this herbal drug as an anti-inflammatory and analgesic agent [7]. Harpagoside is thought to be the active principle and may represent potential anti-inflammatory drugs [8]. In this study, the anti-inflammatory activities of the main component of aqueous extracts from the *Radix Scrophulariae*, harpagoside, was evaluated in vitro to investigate their suppressive effect on the hypoxia-induced microglial cells activation.

## Methods

### Preparation of microglial cells culture

This study received prior approval of the Taipei Medical University Investigation Research Board Committee. Enriched microglial cells culture were derived from the ICR mice neonatal cortex (3–4 days old) using a technique modified from Saura et al. that favors the survival and proliferation of glial cells over neurons [9]. Briefly, mixed glial cultures were prepared from mechanical and chemical dissociation, cortical cells were seeded in DMEM (Life Technologies Inc., Gibco/BRL Division, Grand Island, New York, USA) with 10 % FBS (Life Technologies Inc., Gibco/BRL Division, Grand Island, New York, USA) at a density of 250,000 cells/ml (=62,500 cells/cm^2^). Cells were cultured at 37 °C in humidified 5 % CO_2_/95 % air. Medium was changed every 3 days and confluence of microglial cells culture was achieved after 10–14 days in vitro. Then, microglial cells culture was prepared by mild trypsinization (0.05 % trypsin + 0.2 mM EDTA) and shaking. High purity of microglia can be isolated by shaking off loosely adherent cells (astrocytes) from mixed glial cultures. For the following study, the microglial cells were grown in DMEM supplemented with 10 % fetal bovine serum. The surgical procedures were carried out in accordance with the Declaration of Helsinki and experimental protocols were approved and under supervision by the Medical College’s Animal Research Committee of the Taipei Medical University.

### The effects of harpagoside under hypoxic conditions

The incubation under hypoxic (1 % O_2_) or normoxic conditions was performed with CO_2_/Tri-gas incubator (ATC-SMA-30D, Astec, Osaka, Japan). To evaluate the effects of harpagoside (Biotic Chemical, Lu Chou, New Taipei City, Taiwan) under hypoxic conditions, the microglial cells were pre-incubated with harpagoside for 1 h before hypoxic test.

### 3-[4,5-dimethylthiazol]-2,5-diphenylterazolium bromide assay (MTT assay)

Microglial cells (1 × 10^4^ cells/well) were seeded into eight 96-well plates. After 2 days of incubation, harpagoside at concentrations ranging from 10^−5^ M to 10^−9^ M was added. The 3-(4, 5-dimethylthiazolyl-2)-2, 5-diphenyltetrazolium bromide (MTT; Sigma Co., St. Louis, MO, USA) assay for cell viability was performed at the 1^st^, 3^rd^, 7^th^ day of culture. During the experiment, the treatment (including medium and medication) was changed every 3 days and fresh harpagoside was added at each media change. The level of mitochondrial activity of the microglial cells after harpagoside treatments were determined by colorimetric assay, which detects the conversion of MTT to insoluble formazan. The plates were read on the ELISA reader (Spectra max 340, molecular Devices; CA, USA) at a wavelength of 595 nm.

### RNA extraction, cDNA synthesis, reverse transcriptase polymerase chain reaction (RT-PCR) and quantitative real-time PCR

After treatment, RNA for analysis was isolated at different time points. Briefly, the cell cultures were washed with PBS, total RNA was extracted and then cDNA reversely transcripted. PCR amplification was performed by the Light Cycler FastStart DNA Master SYBR Green I (Roche, Mannheim, Germany). The amplification was performed in a Roche Light Cycler 2.0 instrument under the following condition: initial denaturation at 95 °C for 10 min, 45 cycles of denaturation at 95 °C for 5 s, annealing at 55 °C for 5 s, and extension at 72 °C for 8 s. For each genes analysis, the experiments were repeated at four times. The internal standard gene used was α-tubulin and the analyzed genes were listed in Table 1.

**Table 1:** Primers sequences for reverse transcription–polymerase chain reaction (RT–PCR)

Gene name	Primer	Product (bp)
(Gene bank)	(F:sense primer/R: antisense primer)	
IL-1β	F: 5′- GTGTGTGACGTTCCCATTAGA -3′	101
(NM8361)	R: 5′- AGGTGGAGAGCTTTCAGCTCA -3′	
IL-6	F: 5′- CAAGTCGGAGGCTTAAAC -3′	101
(X54542)	R: 5′- AAGTGCATCATCGTTGTTCAT -3′	
TNF-α	F: 5′- TCTCTACCTTGTTGCCTCCTCTTTT -3′	150
(NM013693.1)	R: 5′- TGTAGGGCAATTACAGTCACGG -3′	
COX-2	F: 5′- CTGACCCCCAAGGCTCCA -3′	94
(NM011198)	R: 5′- CCAGGTCCTCGCTTATGA -3′	
iNOS	F: 5′- AACATCAGGTCGGCCATCA -3′	94
(NM010927)	R: 5′- CGTACCGGATGAGCTGTCA -3′	
β-miroglobulin	F: 5′-TTCAGTGTGAGCCAGGATATAGAAA-3′	153
(NM009735.3)	R: 5′-GAAGCCGAACATACTGAACTGCT-3′	

### Measurement of NO production

Nitrite production in the culture medium was measured of NO production using nitrate/nitrite colorimetric assay kit by the Griess reaction. Briefly, 30 μl culture medium was incubated with 15 μl of 1 % sulfanilamide (Sigma Chemical, St. Louis, MO, USA) in 5 % phosphoric acid for 5 min incubation at 37 °C, then added 15 μl of 0.1 % N-1-naphthyl-ethylenediamine dihydrochloride (Sigma Chemical, St. Louis, MO, USA) in 5 % phosphoric acid. After a 10 min incubation period at 37 °C, absorbance was measured at 550 nm at 37 °C against a blank prepared with 60 μl of distilled water [10].

### Western blot analysis

After treatments, culture medium was discarded and cells were lysed with Laemmli buffer (4 % SDS, 20 % glycerol, 10 % 2-mercaptoethanol, 0.004 % bromophenol blue, 0.125 M Tris/HCl, pH 6.8). Lysates were boiled for 3 min, resolved by 10 % SDS/PAGE using Tris-glycine pH 8.3 (25 mM Tris, 192 mM glycine, 0.1 % SDS) as running buffer, and then electroblotted onto a Hybond nitrocellulose membrane (transfer buffer: 25 mM Tris, 195 mM glycine, 0.05 % SDS, pH 8.3, and 20 % v/v methanol). Non-specific binding sites on the membrane were blocked (1 h, 25 °C) using 5 % ECL membrane blocking agent in Tris-buffered saline (25 mM Tris, 137 mM NaCl, 3 mM KCl, pH 7.4) containing 0.1 % Tween-20 (TBS-Tween). Blots were briefly rinsed with two changes of TBS-Tween buffer, and then washed three times for 10 min each. Afterwards, blots were incubated with an appropriate dilution of specific antibodies for 1 h at 4 °C, washed three times for 10 min each with TBS-Tween, and probed using a 1:1000 dilution of either anti-mouse or anti-rabbit horseradish peroxidase-conjugated antibodies for 1 h at 25 °C [11]. After washing, blots were incubated with the enhanced chemiluminiscence substrate (ECL kit) and the bands were detected using Fujifilm Intelligent Dark Box II equipment coupled to an LAS-1000 digital camera (Valhalla, NY, USA). IMAGE READER LAS-1000 and LPROCESS V1.Z2 software were used to visualize the bands.

### Statistical analysis

All experiments were performed at four times. Results were expressed as mean ± standard deviation of these expe"riments and statistically analyzed by Two-way ANOVA. Statistical significance by Dunnett’s test was set at *p* < 0.05 between the means of the control and test groups.

## Results

### Harpagoside do not affect microglial cells viability

**Fig. 1:**
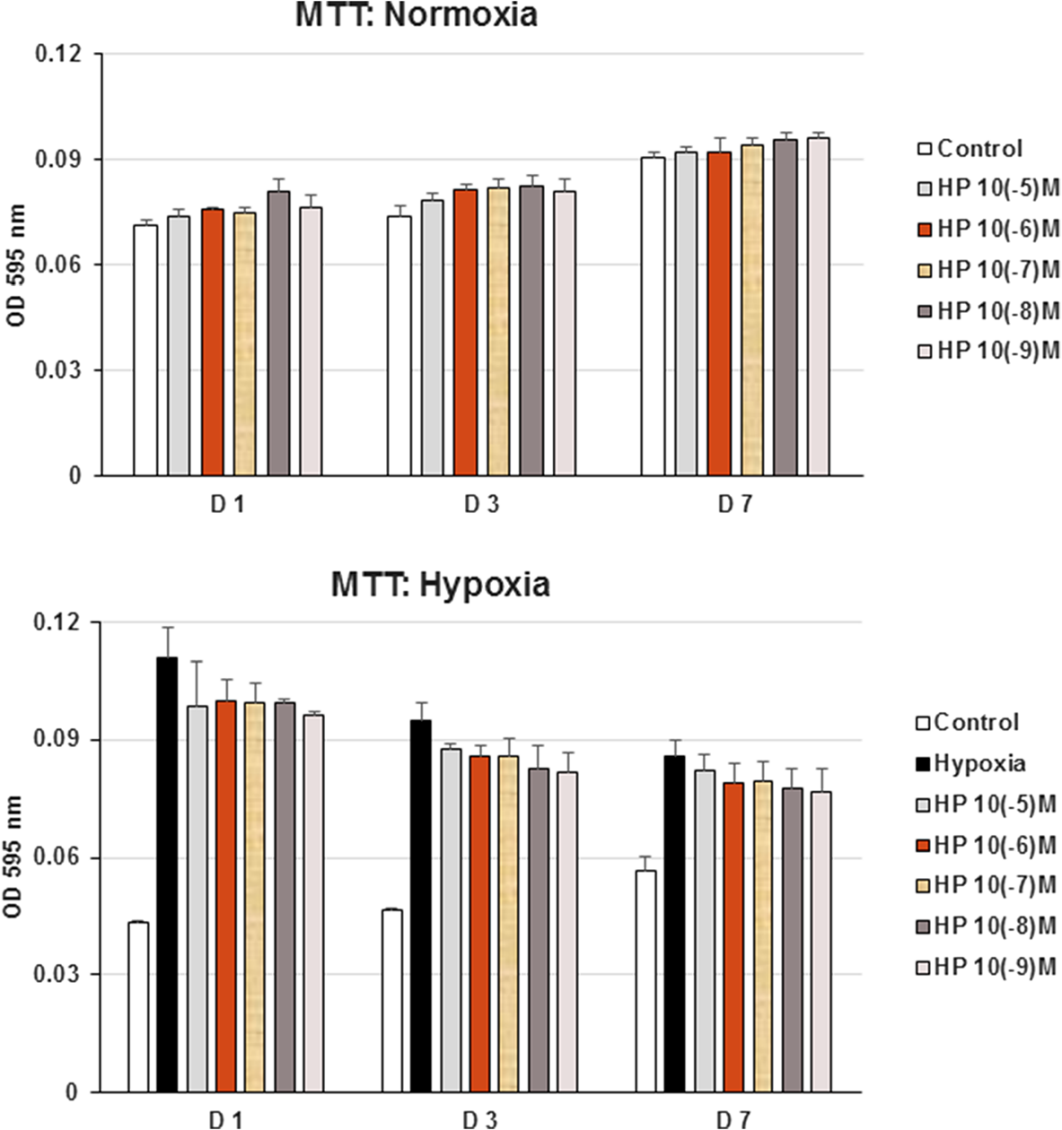
Effect of harpagoside on microglial cells viability and scavenger effect on hypoxia-enhanced microglial cells activation. From this study, harpagoside has no significant cytotoxicity on microglial cells; while there is significant difference observed between treated cells (10^−5^, 10^−6^, 10^−7^, 10^−8^, and 10^−9^ M harpagoside) with that of the control (*n* = 4; *p* < 0.05) at 1, 3, and 7 days’ culture. We chose 10^−8^ M harpagoside for the further evaluation because there was maximal viability of microglial cells at this concentration (112.9 % of control, *p* < 0.01) at the 1st day’s culture. Under hypoxia condition (1 % O_2_), active proliferation of microglial cells was observed when compared with that of normoxic control (*n* = 4; *p* < 0.05). With 1 h pre-incubation with harpagoside, hypoxia-enhanced active proliferation of microglial cells was scavenged (*n* = 4). Similar to previous results, we chose 10^−8^ M harpagoside for the further evaluation because there was persistent effect throughout the 1^st^ day’s culture

The effect of harpagoside on microglial cells viability was examined at harpagoside concentrations of 0, 10^−5^, 10^−6^, 10^−7^, 10^−8^, and 10^−9^ M at 1, 3, and 7 days of culture (*n* = 4; Fig. 1). In this study, there is significant difference observed between treated cells and that of the control (*p* < 0.05). We chose 10^−8^ M harpagoside for further evaluation because there was maximal viability of microglial cells at this concentration (112.9 % of control, *p* < 0.01) at the 1^st^ day’s culture.

### Harpagoside scavenge hypoxia-enhanced microglial cells active proliferation

Under hypoxia condition (1 % O_2_), active proliferation of microglial cells was observed when compared with that of normoxic control (*n* = 4; Fig. 1). With 1 h pre-incubation with harpagoside, hypoxia-enhanced active proliferation of microglial cells was scavenged (*n* = 4; Fig. 1).

### Harpagoside scavenge hypoxia-enhanced microglial cells activation

**Fig. 2:**
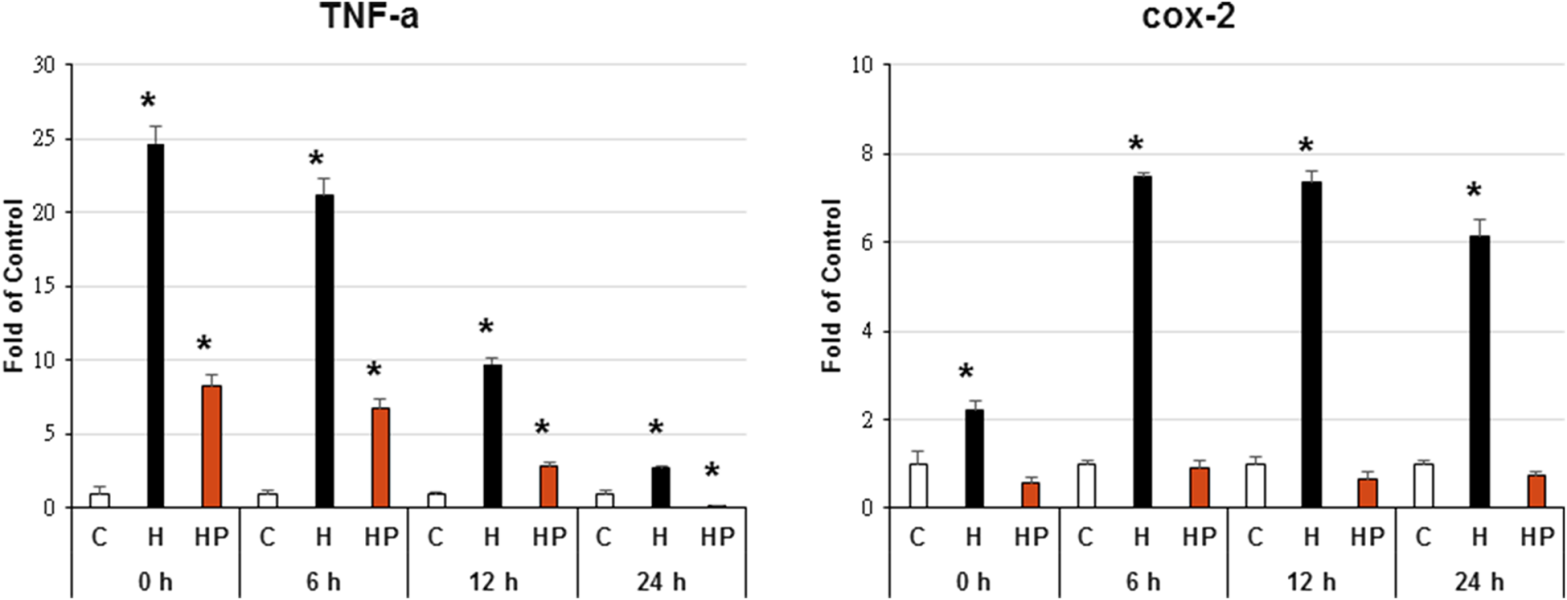
Effect of harpagoside on hypoxia-induced inflammatory gene expression (TNF-α, COX-2) of microglial cells. Three hours’ hypoxia stimulates microglial cells TNF-α and COX-2 genes expression. The maximal effect on COX-2 gene occurred at 6 h’ culture; while for the TNF-α gene, its maximal effect was at 0 h’ culture. The pre-incubation of 10^−8^ M harpagoside again can inhibit these effects (*n* = 4). Note: *: *p* < 0.05

**Fig. 3:**
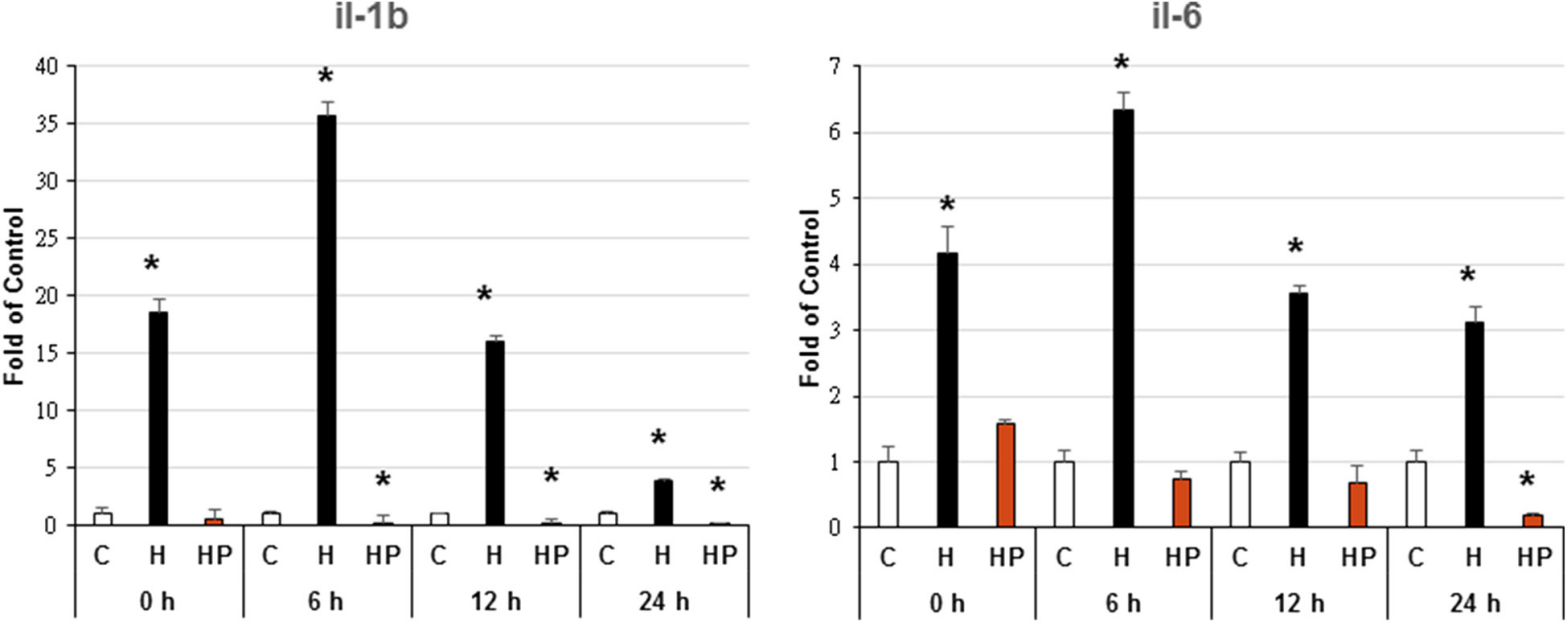
Effect of harpagoside on hypoxia-induced inflammatory gene expression (IL-1β, IL-6) of microglial cells. Three hours’ hypoxia stimulates microglial cells IL-1β and IL-6 genes expression, with its maximal effect occurred at 6 h’ culture. The pre-incubation of 10^−8^ M harpagoside again can inhibit these effects (*n* = 4). Note: *: *p* < 0.05

Three hours’ hypoxia stimulates microglial cells to up-regulate their cyclooxygenase-2 (COX-2), interleukins (IL-1β and IL-6) genes expression, with its maximal effect occurred at 6 h’ culture; while for the tumor necrosis factor (TNF-α) gene, its maximal effect was at 0 h’ culture. The pre-incubation of 10^−8^ M harpagoside again can inhibit these effects (Figs. 2 and 3).

### Harpagoside scavenge hypoxia-enhanced NO synthesis of microglial cells

**Fig. 4:**
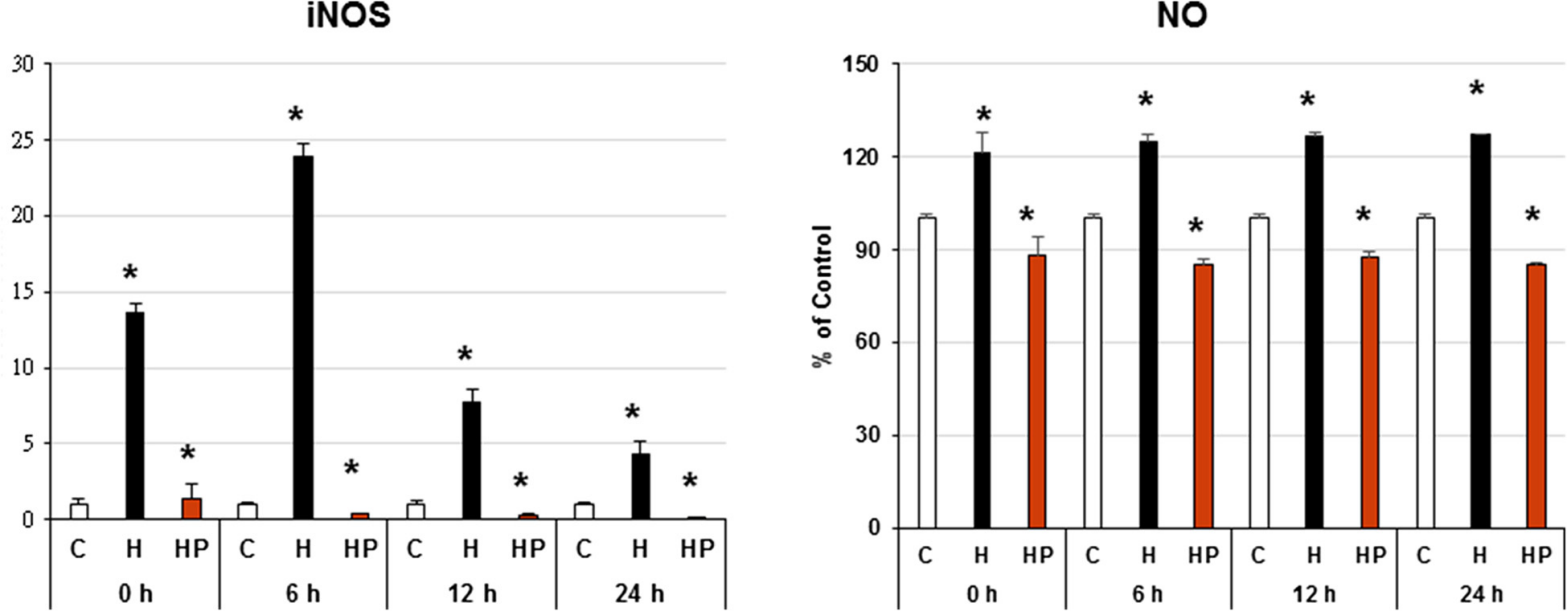
Effect of harpagoside on iNOS gene expression and NO synthesis of microglial cells. Three hours’ hypoxia stimulates microglial cells iNOS gene expression; with its maximal effect occurred at 6 h’ culture; the pre-incubation of harpagoside can down-regulate hypoxia-induced iNOS gene expression. Besides, we found that when microglial cells cultured under hypoxia condition (1 % O_2_), active synthesis of NO was observed; while 1 h pre-incubation with harpagoside on microglial cells does reduce the NO production by microglial cells under hypoxic condition (*n* = 4). Note: *: *p* < 0.05

Three hours’ hypoxia stimulates microglial cells iInducible nitric oxide synthase (iNOS) gene expression; iNOS gene expression significantly upregulated with its maximal effect occurred at 6 h’ culture (up to 23.9 folds of the control). The pre-incubation of harpagoside can down-regulate hypoxia-induced iNOS gene expression with its maximal effect occurred at 24 h’ culture (down to 0.1 folds of the control) (Fig. 4). Besides, we found that when microglial cells cultured under hypoxia condition (1 % O_2_), active synthesis of NO was observed, this attained its maximal effect at 24 h’ culture (up to 127.2 % of the control). While 1 h pre-incubation with harpagoside on microglial cells does reduce the NO production by microglial cells under hypoxic condition, which attained its maximal effect at 24 h’ culture (down to 85.5 % of the control) (*n* = 4; Fig. 4).

### Harpagoside suppress hypoxia-induced microglial cells activation

**Fig. 5:**
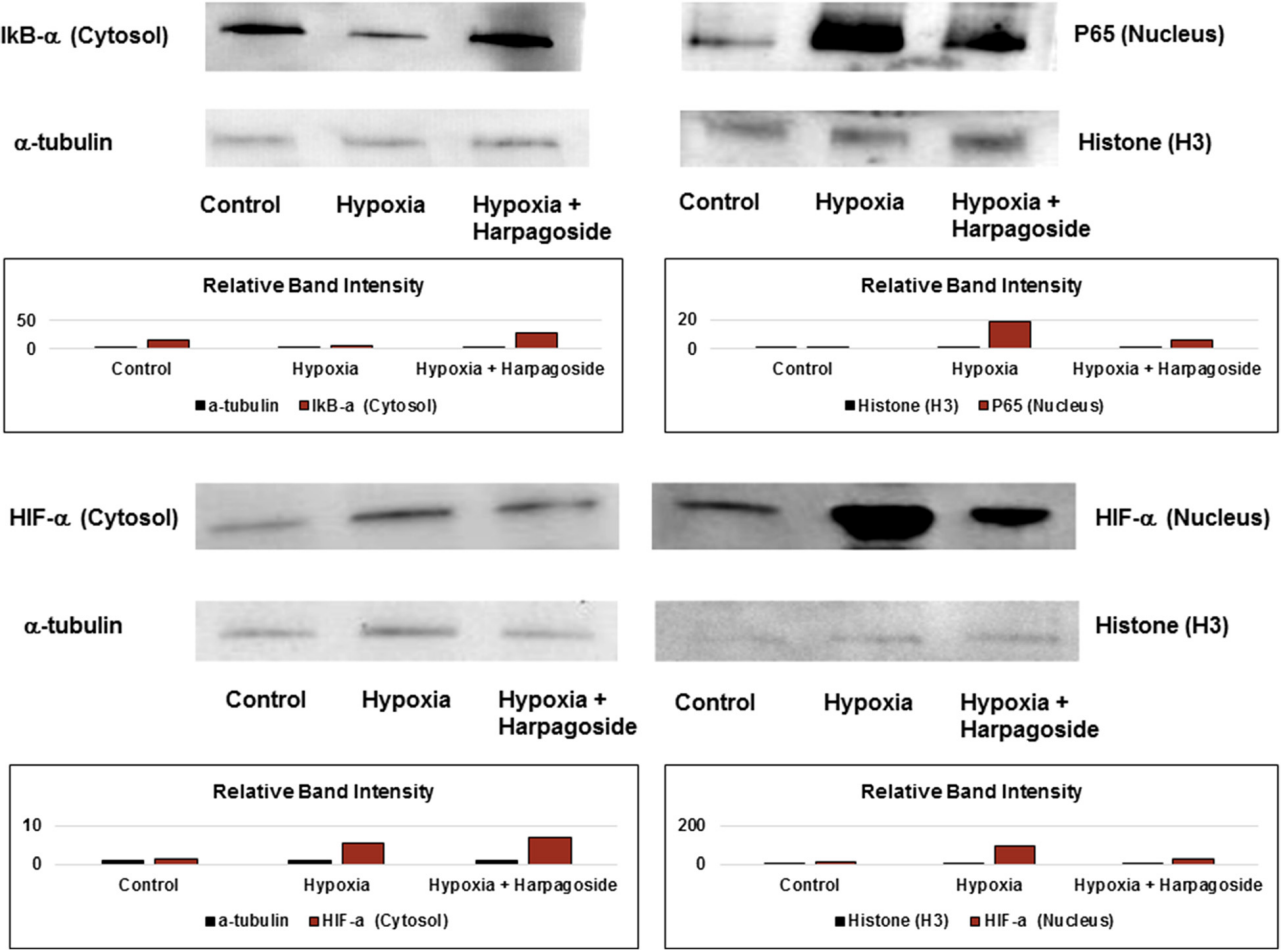
Harpagoside protect against hypoxia-induced toxicity on microglial cells through HIF-α pathway: Western blot quantification. Under 3 h’ hypoxic stimulation, the p65 content within nucleus significantly increased; while the cytosol IκB-α content decreased; theses effect can be reversed by 1 h’s pre-incubation of 10^−8^ M harpagoside. For the HIF-α, hypoxic stimulation can significantly enhance the nuclear HIF-α presentation; while there was no significant effect on cytosol HIF-α; theses effect can be reversed by 1 h’s pre-incubation of 10^−8^ M harpagoside. Intensity of individual bands was quantified using NIH ImageJ densitometry software, and expressed relative to β-actin/histone (H3) signal, as a measure of protein relative abundance in the different samples

Under 3 h’ hypoxic stimulation, the nuclear content of transcription factor p65 (RELA) (p65) and hypoxia-inducible factors (HIF-α) significantly increased; while the cytosol IκB-α content significantly decreased and the cytosol HIF-α content remained stationary. These effects can be reversed by 1 h’s pre-incubation of 10^−8^ M harpagoside (Fig. 5).

## Discussion

During cerebral ischemia, exposure to hypoxia induced microglia activation; hypoxia may not only directly damage neurons, but also promote neuronal injury indirectly via microglia activation. Thus, toxic inflammatory mediators produced by activated microglia may exacerbate neuronal injury following cerebral ischemia [12]. An important factor in the onset of inflammatory process is the overexpression of interleukin (IL)-1, which may cause dysfunction and neuronal death in a vicious circle. Other important cytokines are IL-6 and tumor necrosis factor (TNF)-α. Thus, therapeutic strategies directed at controlling the activation of microglia and astrocytes and the excessive production of pro-inflammatory and pro-oxidant factors may be valuable to control neurodegeneration in AD and dementia [13].

TNF-α plays a central role in the cytokine cascade during an inflammatory response. Neuronal production of TNF-α has also been demonstrated [14]. In the central nervous system (CNS), tumor necrosis factor-alpha (TNF-α) plays a critical role as an inflammatory mediator. There exist a positive feedback loop in the activation of microglia via TNF-alpha; this may be involved in the prolonged activation of microglia [15]. Inflammation induced by the generation of prostanoids may well contribute to neuronal destruction. Although COX-2 expression is driven by physiological synaptic activity [16] and therefore may be regarded as physiologically expressed protein in a subclass of neurons; in this study, we demonstrated that 3 h’ hypoxia stimulates microglial cells to up-regulate their TNF-α and COX-2 genes expression; while the pre-incubation of 10^−8^ M harpagoside again can scavenge these effects (Fig. 2).

Pharmacological evaluations have proven the effectiveness of harpagoside as an anti-inflammatory and analgesic agent. It inhibit the cellular expression of cyclooxygenase-2 and inducible nitric oxide by suppression of NF-kappaB activation, thereby inhibit downstream inflammation and subsequent pain events [7, 17]. IL-1 is an important initiator of the immune response, playing a key role in the onset and development of a complex hormonal and cellular inflammatory cascade. Elevated IL-1β has been detected in the CSF and brain parenchyma within the early hours after brain injury in both humans and rodents [18]. IL-1 has also been documented to play a role in neuronal degeneration. In astrocytes, IL-1 induces IL-6 production, stimulates iNOS activity [19], and induces the production of macrophage colony-stimulating factor (M-CSF). In addition, IL-1 enhances microglial activation and additional IL-1 production, astrocyte activation, thereby establishing a self-propagating cycle [20]. IL-6 is a multifunctional cytokine that plays an important role in host defense, with major regulatory effects upon the inflammatory response [21]. IL-6 belongs to the neuropoietin family of cytokines, and it has both direct and indirect neurotrophic effects on neurons [22]. IL-6 promotes astrogliosis, activates microglia, and stimulates the production of acute phase proteins [23]. In this study, 3 h’ hypoxia stimulates microglial cells to up-regulate their IL-1β and IL-6 genes expression, with its maximal effect occurred at 6 h’ culture. The pre-incubation of 10^−8^ M harpagoside again can inhibit these effects (Fig. 3).

Nitric oxide (NO) is a molecule with pleiotropic effects in different tissues. NO is a well-known vasorelaxant agent, but it works as a neurotransmitter when produced by neurons and is also involved in defense functions when it is produced by immune and glial cells [24]. Glial- and neuronal-derived iNOS-related long-term NO release and NO-dependent peroxynitrite formation have been demonstrated to cause neuronal dysfunction and cell death in vitro and in vivo [25–27]. Under pathophysiological conditions, NO has damaging effects. In disorders involving oxidative stress, NO increases cell damage through the formation of highly reactive peroxynitrite [28]. In this study, 3 h’ hypoxia stimulates microglial cells iNOS gene expression and active synthesis of NO; the pre-incubation of harpagoside can down-regulate hypoxia-induced iNOS gene expression and reduces the NO production by microglial cells under hypoxic condition (Fig. 4).

Hypoxia could lead to the inflammatory activation of microglia [29]. During cerebral ischemia, hypoxia may not only directly damage neurons, but also promote neuronal injury indirectly via microglia activation [30]. The hypoxia-inducible factor-1 (HIF-1) is primarily involved in the sensing and adapting of cells to changes in the O_2_ level, which is regulated by many physiological functions. The nuclear factor (NF)-kappaB transcriptional system is a major effector pathway involved in inflammation and innate immune responses. The flavonoid harpagoside is found in various herbal extracts and has shown anti-inflammatory properties. In our study, under 3 h’ hypoxic stimulation, the nuclear HIF-α presentation and the p65 content within nucleus significantly increased; while the cytosol IκB-α content significantly decreased; theses effect can be reversed by 1 h’s pre-incubation of 10^−8^ M harpagoside (Fig. 5). We report that harpagoside significantly blocks hypoxia-induced I-kappa-B phosphorylation/degradation, and NF-kappa-B transcriptional activity in mice microglial cells. Modulation of innate immunity by natural plant products may represent an attractive strategy to prevent inflammation associated with neuron dysfunction mediated by activated microglia [31].

Flavonoids and their polymers constitute a large class of food constituents, many of which alter metabolic processes and have a positive impact on health [32]. Flavonoids are a subclass of polyphenols. Microglia are innate immune cells in the central nervous system. Activated microglia can produce various proinflammatory cytokines and nitric oxide (NO), which may exert neurotoxic effects. To search for the novel therapeutic agents against neuroinflammatory diseases, we have screened a series of flavonoid compounds using a cell-based assay. Our studies showed that harpagoside appear to down-regulate the gene expression of TNF-α, interleukin (IL)-1 beta, cyclooxygenase (COX-2) and inducible nitric oxide synthase (iNOS) at mRNA levels and also may act to inhibit proinflammatory responses [31, 33]. Harpagoside also significantly suppressed I kappa B degradation, nuclear translocation of NF-kappa B, p65 and HIF-1α. These results indicate that harpagoside has a strong anti-inflammatory activity in brain microglia, and could be a potential therapeutic agent for the treatment of neuroinflammatory diseases [33].

Although flavonoid-rich diets and flavonoid administration prevent cognitive impairment associated with inflammation in animal studies [34], dietary bioactives have potential to restore the population of microglial cells in the senescent brain to a more quiescent state because neuroinflammation and cognitive deficits are co-morbid factors in many chronic inflammatory diseases [35]. However, retrospective cohort studies are inconsistent in showing an inverse association between dietary flavonoid intake and dementia or neurodegenerative disease risk in humans [36]. Thus, future human studies (ideally randomized clinical trials) will be required. These studies should involve supplementation with relatively high doses of specific purified flavanoids to shed light to the apparent inverse risk relationship with neurodegenerative diseases and also to determine if such compounds are therapeutically beneficial.

## Conclusions

**Fig. 6:**
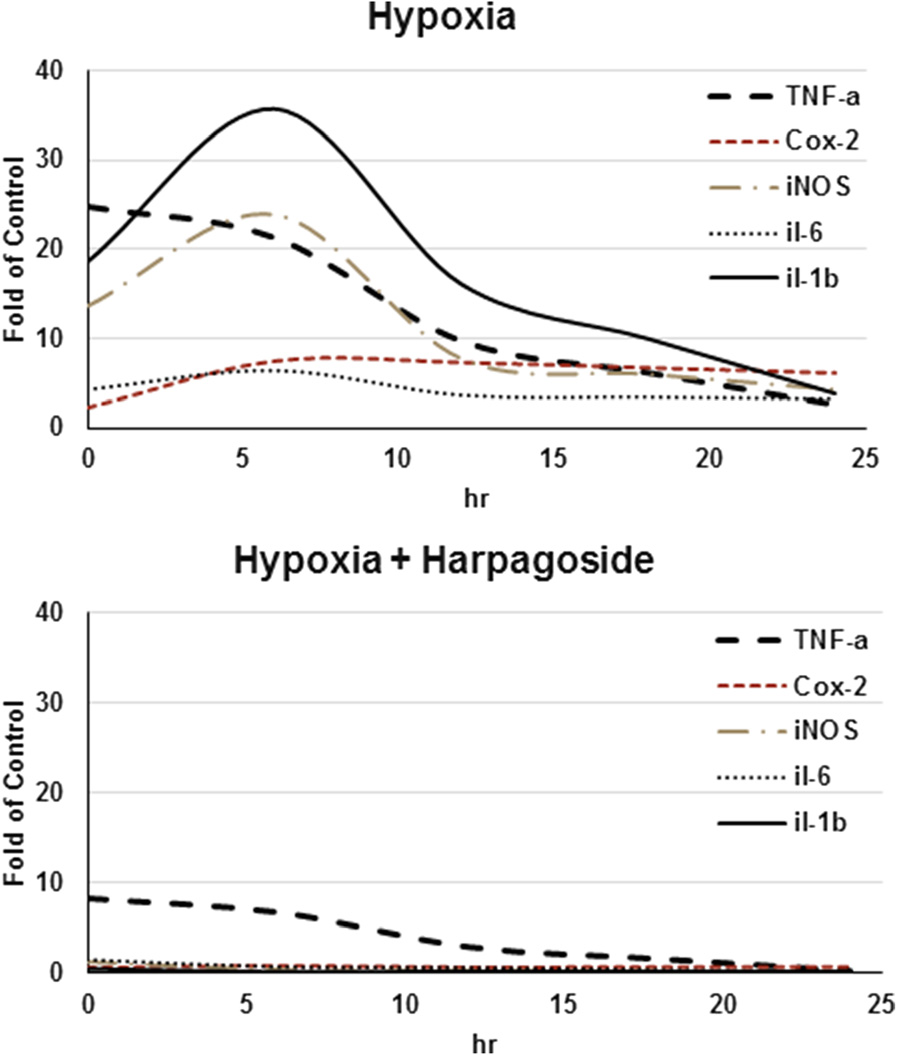
Molecular mechanism of harpagoside on the hypoxia-induced toxicity on microglial cells

Hypoxia occurs when oxygen availability drops below the levels necessary to maintain normal rates of metabolism. Because of its high metabolic activity, the brain is highly sensitive to hypoxia. Hypoxia could lead to microglia activation and toxic inflammatory mediators’ overproduction. Those inflammatory molecules could amplify the neuroinflammatory process and exacerbate neuronal injury. In this study, we found that harpagoside could suppress the microglial TNF-α, IL-1β, iNOS, COX-2 and IL-6 mRNA expression and lower microglia nitric oxide production. The above experimental data indicated that harpagoside could reduce microglia inflammatory mediators’ overproduction. In addition, the anti-inflammatory mechanism of harpagoside might be associated with the transcription factor nuclear factor kappa B (NF-κB) signaling pathway. Harpagoside could decrease IκB-α protein phosphorylation and inhibit p65 protein translocation from the cytosol to the nucleus, thus suppress NF-κB activation and reduce the HIF-1α generation (Fig. 6).

In vitro screening for bioactive components is well established as a means for searching potential candidates in vivo. However, these predictions are based on observation from in vitro experiments, some un-perceived mistakes (such as commonly encountered uncertainties and sources of bias and error) of a compound can lead to an inaccurate prediction of an in vivo interaction. The results from this in vitro study is still provocative and too few to draw definitive conclusions. Further supporting data from many in vitro mechanistic studies and in vivo studies will convincingly show their potential effects of this bioactive molecule and then sufficiently tantalize to justify large-scale clinical intervention studies. This intervention, however, is important because we do contribute a major new insights into the molecular mechanisms that regulate the oxygen-sensitivity of HIF, and in the development of compounds with which to manipulate HIF activity, are forcing serious consideration of HIF as a therapeutic target for diverse CNS disorders associated with hypoxia [37]. However, the mechanisms of harpagoside have not been completely understood and the facts need to be validated by further studies both in vitro and in vivo.

## References

[1] Li Y, Chen G, Zhao J, Nie X, Wan C, Liu J (2013). 2,3,7,8-Tetrachlorodibenzo-p-dioxin (TCDD) induces microglial nitric oxide production and subsequent rat primary cortical neuron apoptosis through p38/JNK MAPK pathway. Toxicology.

[2] Yang J, Wang C, Nie X, Shi S, Xiao J, Ma X (2015). Perfluorooctane sulfonate mediates microglial activation and secretion of TNF-αthrough Ca2+−dependent PKC-NF-кB signaling. Int Immunopharmacol.

[3] Garden GA, Möller T (2006). Microglia biology in health and disease. J Neuroimmune Pharmacol.

[4] Kaindl AM, Degos V, Peineau S, Gouadon E, Chhor V, Loron G (2012). Activation of microglial N-methyl-D-aspartate receptors triggers inflammation and neuronal cell death in the developing and mature brain. Ann Neurol.

[5] Rubio-Perez JM, Morillas-Ruiz JM (2012). A review: inflammatory process in Alzheimer’s disease, role of cytokines. Sci World J.

[6] Peng L, Li M, Xu YZ, Zhang GY, Yang C, Zhou YN (2012). Effect of Si-Miao-Yong-An on the stability of atherosclerotic plaque in a diet-induced rabbit model. J Ethnopharmacol.

[7] Huang TH, Tran VH, Duke RK, Tan S, Chrubasik S, Roufogalis BD (2006). Harpagoside suppresses lipopolysaccharide-induced iNOS and COX-2 expression through inhibition of NF-kappa B activation. J Ethnopharmacol.

[8] Kaszkin M, Beck KF, Koch E, Erdelmeier C, Kusch S, Pfeilschifter J (2004). Downregulation of iNOS expression in rat mesangial cells by special extracts of Harpagophytum procumbens derives from harpagoside-dependent and independent effects. Phytomedicine.

[9] Saura J, Tusell JM, Serratosa J (2003). High-yield isolation of murine microglia by mild trypsinization. Glia.

[10] Green LC, Wagner DA, Glogowski J, Skipper PL, Wishnok JS, Tannenbaum SR (1982). Analysis of nitrate, nitrite, and [15N]nitrate in biological fluids. Anal Biochem.

[11] Vittori D, Pregi N, Pérez G, Garbossa G, Nesse A (2005). The distinct erythropoietin functions that promote cell survival and proliferation are affected by aluminum exposure through mechanisms involving erythropoietin receptor. Biochim Biophys Acta.

[12] Kaur C, Rathnasamy G, Ling EA (2013). Roles of activated microglia in hypoxia induced neuroinflammation in the developing brain and the retina. J Neuroimmune Pharmacol.

[13] Agostinho P, Cunha RA, Oliveira C (2010). Neuroinflammation, oxidative stress and the pathogenesis of Alzheimer’s disease. Curr Pharm Des.

[14] Perry RT, Collins JS, Wiener H, Acton R, Go RC (2001). The role of TNF and its receptors in Alzheimer’s disease. Neurobiol Aging.

[15] Kuno R, Wang J, Kawanokuchi J, Takeuchi H, Mizuno T, Suzumura A (2005). Autocrine activation of microglia by tumor necrosis factor-alpha. J Neuroimmunol.

[16] Yermakova A, O’Banion MK (2000). Cyclooxygenases in the central nervous system: implications for treatment of neurological disorders. Curr Pharm Des.

[17] Háznagy-Radnai E, Balogh Á, Czigle S, Máthé I, Hohmann J, Blazsó G (2012). Antiinflammatory activities of Hungarian Stachys species and their iridoids. Phytother Res.

[18] Winter CD, Iannotti F, Pringle AK, Trikkas C, Clough GF, Church MK (2002). A microdialysis method for the recovery of IL-1beta, IL-6 and nerve growth factor from human brain in vivo. J Neurosci Methods.

[19] Rossi F, Bianchini E (1996). Synergistic induction of nitric oxide by beta-amyloid and cytokines in astrocytes. Biochem Biophys Res Commun.

[20] Mrak RE, Griffin WS (2001). Interleukin-1, neuroinflammation, and Alzheimer’s disease. Neurobiol Aging.

[21] Raivich G, Bohatschek M, Kloss CU, Werner A, Jones LL, Kreutzberg GW (1999). Neuroglial activation repertoire in the injured brain: graded response, molecular mechanisms and cues to physiological function. Brain Res Brain Res Rev.

[22] Benveniste EN (1998). Cytokine actions in the central nervous system. Cytokine Growth Factor Rev.

[23] Heyser CJ, Masliah E, Samimi A, Campbell IL, Gold LH (1997). Progressive decline in avoidance learning paralleled by inflammatory neurodegeneration in transgenic mice expressing interleukin 6 in the brain. Proc Natl Acad Sci U S A.

[24] Heneka MT, Wiesinger H, Dumitrescu-Ozimek L, Riederer P, Feinstein DL, Klockgether T (2001). Neuronal and glial coexpression of argininosuccinate synthetase and inducible nitric oxide synthase in Alzheimer disease. J Neuropathol Exp Neurol.

[25] Smith MA, Richey Harris PL, Sayre LM, Beckman JS, Perry G (1997). Widespread peroxynitrite-mediated damage in Alzheimer’s disease. J Neurosci.

[26] Boje KM, Arora PK (1992). Microglial-produced nitric oxide and reactive nitrogen oxides mediate neuronal cell death. Brain Res.

[27] Heneka MT, Löschmann PA, Gleichmann M, Weller M, Schulz JB, Wüllner U (1998). Induction of nitric oxide synthase and nitric oxide-mediated apoptosis in neuronal PC12 cells after stimulation with tumor necrosis factor-alpha/lipopolysaccharide. J Neurochem.

[28] Guix FX, Uribesalgo I, Coma M, Muñoz FJ (2005). The physiology and pathophysiology of nitric oxide in the brain. Prog Neurobiol.

[29] Eltzschig HK, Carmeliet P (2011). Hypoxia and inflammation. N Engl J Med.

[30] Lu DY, Liou HC, Tang CH, Fu WM (2006). Hypoxia-induced iNOS expression in microglia is regulated by the PI3-kinase/Akt/mTOR signaling pathway and activation of hypoxia inducible factor-1alpha. Biochem Pharmacol.

[31] Kim JS, Jobin C (2005). The flavonoid luteolin prevents lipopolysaccharide-induced NF-kappaB signalling and gene expression by blocking IkappaB kinase activity in intestinal epithelial cells and bone-marrow derived dendritic cells. Immunology.

[32] Vogiatzoglou A, Mulligan AA, Lentjes MA, Luben RN, Spencer JP, Schroeter H (2015). Flavonoid intake in European adults (18 to 64 years). PLoS One.

[33] Zheng LT, Ock J, Kwon BM, Suk K (2008). Suppressive effects of flavonoid fisetin on lipopolysaccharide-induced microglial activation and neurotoxicity. Int Immunopharmacol.

[34] Rinwa P, Kumar A (2013). Quercetin suppress microglial neuroinflammatory response and induce antidepressent-like effect in olfactory bulbectomized rats. Neuroscience.

[35] Johnson RW (2015). Feeding the beast: can microglia in the senescent brain be regulated by diet?. Brain Behav Immun.

[36] Engelhart MJ, Geerlings MI, Ruitenberg A, van Swieten JC, Hofman A, Witteman JC (2002). Dietary intake of antioxidants and risk of Alzheimer disease. JAMA.

[37] Freeman RS, Barone MC (2005). Targeting hypoxia-inducible factor (HIF) as a therapeutic strategy for CNS disorders. Curr Drug Targets CNS Neurol Disord.

